# The agglomeration state of nanoparticles can influence the mechanism of their cellular internalisation

**DOI:** 10.1186/s12951-017-0281-6

**Published:** 2017-06-26

**Authors:** Blanka Halamoda-Kenzaoui, Mara Ceridono, Patricia Urbán, Alessia Bogni, Jessica Ponti, Sabrina Gioria, Agnieszka Kinsner-Ovaskainen

**Affiliations:** European Commission Joint Research Centre, Directorate for Health, Consumers and Reference Materials, Via E. Fermi 2749, TP 127, 21027 Ispra, VA Italy

**Keywords:** Silica nanoparticles, Cell uptake, Endocytosis route, Agglomeration/aggregation, In vitro

## Abstract

**Background:**

Significant progress of nanotechnology, including in particular biomedical and pharmaceutical applications, has resulted in a high number of studies describing the biological effects of nanomaterials. Moreover, a determination of so-called “critical quality attributes”, that is specific physicochemical properties of nanomaterials triggering the observed biological response, has been recognised as crucial for the evaluation and design of novel safe and efficacious therapeutics. In the context of in vitro studies, a thorough physicochemical characterisation of nanoparticles (NPs), also in the biological medium, is necessary to allow a correlation with a cellular response. Following this concept, we examined whether the main and frequently reported characteristics of NPs such as size and the agglomeration state can influence the level and the mechanism of NP cellular internalization.

**Results:**

We employed fluorescently-labelled 30 and 80 nm silicon dioxide NPs, both in agglomerated and non-agglomerated form. Using flow cytometry, transmission electron microscopy, the inhibitors of endocytosis and gene silencing we determined the most probable routes of cellular uptake for each form of tested silica NPs. We observed differences in cellular uptake depending on the size and the agglomeration state of NPs. Caveolae-mediated endocytosis was implicated particularly in the internalisation of well dispersed silica NPs but with an increase of the agglomeration state of NPs a combination of endocytic pathways with a predominant role of macropinocytosis was noted.

**Conclusions:**

We demonstrated that the agglomeration state of NPs is an important factor influencing the level of cell uptake and the mechanism of endocytosis of silica NPs.

**Electronic supplementary material:**

The online version of this article (doi:10.1186/s12951-017-0281-6) contains supplementary material, which is available to authorized users.

## Background

A detailed understanding of the mechanisms of interaction between engineered nanoparticles (NPs) and biological systems is essential to properly assess the safety of newly developed nanotechnological and nanomedicinal products. Upon exposure, NPs may interact with the outer surface of the cellular membrane and subsequently enter the cells by different endocytic routes. Elucidation of the mechanism by which NPs are internalized into the cells can provide insights about the intracellular trafficking, fate and cytotoxic profile of NPs [1]. Targeting of specific cellular structures, the release of NPs by the cells or, on the contrary, their degradation in lysosomes are all key features that can significantly affect the NPs toxicity/safety, but also the efficacy of novel nanomedicines.

All these processes are highly dependent on the mechanism of NP internalisation [2, 3]. The involvement of various endocytic pathways in NP uptake has been investigated in several in vitro studies, and the obtained results show wide variability with respect to cell lines and nanoparticles used [4–6].

It is well recognised that an accurate characterisation of NPs in the biological medium and under the conditions of an in vitro study is necessary to properly interpret the results and to enable correlation of the physicochemical parameters with the biological response [7]. Indeed, NP properties such as size, shape, surface chemistry and charge were shown to affect the level of cellular uptake and the mechanism of the endocytosis [8–11]. However up to date, little attention has been paid to the question of how this process is influenced by the agglomeration state of NPs. Though, the ability to agglomerate is one of the predominant features of a NP suspension. Changes to the pH and ionic strength or the presence of biomolecules, particularly proteins, can easily modify the NP surface properties, leading to the loss of colloidal stability and formation of agglomerates. That is why this study focused on understanding how the agglomeration state of NPs can influence the endocytic mechanism by which NPs can enter the cells. We have observed in our previous study [7] that silicon dioxide NPs showed a time-dependent tendency to agglomerate in the complete medium of CaCo-2 cells. Therefore, in this study we used the same amorphous fluorescent Rubipy-SiO_2_ NPs of two different sizes, 30 and 80 nm that were either freshly dispersed in complete medium and added to the cells, or pre-incubated with the complete medium for 24 h in order to allow the agglomeration. Subsequently, for the agglomerated and non-agglomerated NPs of both sizes we compared the level of cellular uptake and the endocytosis route in Caco-2 cells. We used flow cytometry to quantify the cellular uptake, transmission electron microscopy (TEM) to visualise the NPs inside the cells and a high-throughput fluorescence microscopy technique to colocalise NPs with clathrin, caveolin-1 and SNX5 antibodies. We also performed blocking of different endocytic routes by chemical inhibitors and by gene silencing to assess the effect on NP internalisation.

## Methods

### Nanoparticle synthesis and characterization

The mono-dispersed fluorescent particles of silicon dioxide 30 and 80 nm labelled with Rubipy were synthetized as described previously [7]. Physicochemical characterization of the NPs including size distribution, zeta potential and fluorescence spectrum carried out in water, PBS and cell culture medium was already described [4]. Additionally, the size distribution of Rubipy-SiO_2_ NPs was determined in complete CaCo-2 cell culture medium by centrifugal liquid sedimentation (CLS) in a sucrose density gradient using a CLS Disc Centrifuge model DC24000UHR (CPS Instruments Europe, Netherlands). The samples were prepared as a 1 mg/ml suspension of Rubipy-SiO_2_ NPs in cell culture media (CCM) containing 10% of foetal bovine serum, and the measurements were performed either immediately or after 24 h incubation at 37 °C. In addition, the particle size in complete CaCo-2 cell culture medium was evaluated by TEM. Ultrathin Formvar-coated 200-mesh copper grids (Tedpella Inc.) were first functionalized by placing the carbon-coated side on a drop of 20 μl of Alcian blue (2% in water) deposited on a Parafilm. After 10 min incubation the grid was washed 5 times by the deposition on the drops of water placed on a Parafilm and the excess fluid was removed by blotting its edge on a strip of paper tissue, leaving a rest of humidity. Finally the grid was placed on a 20 μl drop of the corresponding sample, incubated for 10 min and the excess of fluid was removed again with a paper tissue. TEM (JEOL JEM 2100, Japan) at an accelerating voltage of 200 kV was used to visualize the nanoparticles.

Before the biological experiments, Rubipy-SiO_2_ NPs were purified in centrifugal filters (Amicon Ultra, 10K, Milipore, Italy) at 3000 rpm for 10 min at room temperature (RT) and re-suspended in sterile PBS. The fluorescence spectrum of Rubipy-SiO_2_ NPs before and after the centrifugation was compared and used for the calculation of the NP concentration. The fluorescence intensities of different concentrations of Rubipy-SiO_2_ NPs in PBS and in complete CaCo-2 medium without phenol red were obtained using fluorescence spectrophotometer (Cary Eclipse, Varian, Australia Pty Ltd). The scan of the fluorescence emission between 500 and 700 nm was carried out at excitation 460 nm.

### Cell lines and culture conditions

Colorectal adenocarcinoma CaCo-2 cells (ACC169, DSMZ, Braunschweig, Germany) were cultured under standard cell culture conditions in Dulbecco’s modified Eagle’s medium with high glucose (4.5 g/L), supplemented with 10% heat inactivated Foetal Bovine Serum (North American origin), 1% penicillin–streptomycin, 4 mM l-glutamine and 1% non-essential amino acids. All cell culture reagents were purchased from Thermo Fisher Scientific, Italy. The experiments were performed between passages 1–10 after defrosting of cells from liquid nitrogen storage.

### Internalisation of endocytic markers

Cells were seeded in a 24-well plate (Costar, Italy) at a density of 6 × 10^4^ cells/well, grown until 80–90% confluence, washed, and after serum starvation for 2 h, pre-incubated for 30 min with endocytosis inhibitors: chlorpromazine 50 µM, dynasore 80 µM, methyl-beta-cyclodextrin 5 mM, nystatin 40 µg/ml, genistein 200 µM, or EIPA 75 µM (all from Sigma-Aldrich, Italy). The following endocytic markers were then added to the cells for 30 min: Transferrin Alexa Fluor 488 conjugate 50 µg/ml (clathrin-mediated endocytosis), Bodipy-FL C5-Lactosylceramide (LaCer) complexed to bovine serum albumin (BSA) 1 µg/ml (caveolae-mediated endocytosis), and Alexa Fluor 488-labeled Dextran 10 kDa, 200 µg/ml (macropinocytosis) (all from Thermo Fisher Scientific, Italy).

After exposure the cells were washed twice in cold washing buffer (Hepes 10 mM, NaN_3_ 5 mM) and the remaining membrane bound label was removed according to previously published methods [12, 13]. Briefly, 1 min incubation in ice-cold acidic wash (0.2 M acetic acid, 0.2 M NaCl, pH 2.0), followed by two washes in cold washing buffer was employed to remove surface-bound Transferrin. For the LaCer uptake the “back exchange” procedure was applied, consisting on 1 h washing with 5% fatty acid-free BSA in washing buffer at 4 °C changing for fresh BSA every 10 min. After the washing procedure, the cells were detached by trypsinisation and analysed immediately by flow cytometry.

### Analysis of uptake of Rubipy-SiO_2_ NPs by flow cytometry

Cells were seeded in a 24-well plate (Costar) at a density of 6 × 10^4^ cells/well, grown until 80–90% confluence and exposed to 200 µg/ml of 30 and 80 nm Rubipy-SiO_2_ NPs freshly dispersed in complete medium or pre-incubated in complete medium for 24 h at 37 °C. Depending on the purpose of the assay the exposure was done at 37 °C or at 4 °C. For the study of endocytic pathways before the exposure to Rubipy-SiO_2_ NPs, the cells were pre-incubated for 30 min with endocytosis inhibitors, as described above. Following the exposure to Rubipy-SiO_2_ NPs the cells were washed 3 times in PBS, trypsinised and blocked with a complete cell culture medium, washed again in PBS and analysed immediately by flow cytometry.

Evaluation of cell associated fluorescence, forward scattering (FSC) and side scattering (SSC) were carried out using CyFlow space flow cytometer (Partec, Munster, Germany) and the data were analysed using FCS Express 4 software (De Novo, Los Angeles, CA). Laser excitation was 488 nm and emission bandpass wavelength was 590/50 nm for Rubipy-SiO_2_ NPs related fluorescence. A minimum of 15,000 cells per sample were analysed; cells debris, nanoparticles and doublets were excluded from the analysis by gating on the FSC versus SSC log graph and on the FL-2 area versus FL-2 width graph, respectively. The median cell associated fluorescence after the subtraction of cell autofluorescence was averaged between three independent experiments (2 replicas each). The results were then normalised according to the reference values of fluorescence intensity of 30 and 80 nm Rubipy-SiO_2_ NPs to allow the comparison of cell uptake of both sizes of NPs. The efficiency of inhibitors is calculated as a percentage of the uptake by the control cells without any inhibitor.

### Analysis of uptake of Rubipy-SiO_2_ NPs by transmission electron microscopy

Following 3 and 6 h of exposure to 200 µg/ml of 30 and 80 nm Rubipy-SiO_2_ NPs freshly dispersed in complete medium or pre-incubated in complete medium for 24 h at 37 °C, the cells were washed 3 times in PBS, trypsinised, blocked with a complete cell culture medium and washed again 3 times in PBS by centrifugation (200×*g*, 5 min). The supernatant was discarded and the cells were fixed using a Karnovsky 2% v/v solution (glutaraldehyde and paraformaldehyde in 0.05 M cacodylate, pH 7.3, Sigma-Aldrich, Italy) over night. Cells were then washed 3 times with 0.05 M cacodylate, pH 7.3 and post-fixed in osmium tetroxide solution in 0.1 M cacodylate, pH 7.3 (both from Sigma-Aldrich, Italy) for 1 h. After 3 washes in cacodylate 0.05 M of 10 min each, cells were dehydrated in a graded series of ethanol solution in MilliQ water (30; 50; 75; 95% for 15 min each, and 100% for 30 min), incubated in absolute propylene oxide (Sigma-Aldrich, Italy) for 20 min (2 changes of 10 min each) and embedded in a solution of 1:1 epoxy resin (Sigma-Aldrich, Italy) and propylene oxide for 90 min. This mixture was renewed with pure epoxy resin (Sigma-Aldrich, Italy) over night at room temperature and later polymerized at 60 °C for 48 h. Ultrathin sections (60–90 nm) were obtained using Leica UCT ultramicrotome (Leica, Italy) and stained with uranyl acetate for 25 min and lead citrate for 20 min (both from Sigma-Aldrich, Italy), washed and dried. Ultrathin sections were imaged using JEOL-JEM 2100 TEM (JEOL, Milan, Italy) at 120 kV.

### Colocalisation with endocytic proteins

Cells were seeded in a 96-well plate (black, glass bottom, Greiner Bio-one) at a density of 1 × 10^4^ cells/well and grown until 80–90% confluence was reached. Afterwards the cells were exposed to 200 µg/ml of 30 or 80 nm Rubipy-SiO_2_ NPs freshly dispersed in complete medium or pre-incubated in complete medium for 24 h at 37 °C. After 3 h exposure the cells were washed 3 times in PBS, fixed with 3.7% paraformaldehyde in PBS, permeabilised with cold methanol at −20 °C and blocked for 30 min at RT with 3% BSA in PBS. Overnight incubation at 4 °C with primary antibodies: either anti-clathrin heavy chain (Abcam, rabbit polyclonal, 1:2000), anti-caveolin-1 (Abcam, rabbit polyclonal, 1:800) or anti-SNX5 (Abcam, goat polyclonal, 1:90) was followed by 45 min incubation at 37 °C with secondary antibodies: anti-rabbit IgG (Invitrogen, goat, Cy^®^ 5 conjugate, 1:300), or anti-goat IgG (Invitrogen, rabbit, Alexa Fluor^®^ 647, 1:300) and DAPI (Molecular Probes, 2,5 µg/ml final concentration). Cells were imaged using IN Cell Analyzer 2200 (GE Healthcare). During acquisition, a minimum of 20 fields per well were imaged using a 60× objective. The representative images were selected, DAPI, Cy3 and Cy5 channels were merged and the qualitative analysis of colocalisation between images from channel Cy3 (Rubipy-SiO_2_ NPs) and from channel Cy5 (secondary antibodies) was performed using ImageJ software.

### Transfection of cells with si-RNA

The cells were seeded in a 24-well plate (Costar) at a density of 5 × 10^4^ cells/well and cultured for 24 h to be 30–40% confluent on the day of transfection. The si-RNA procedure was performed according to the manufacturer's protocol using Silencer^®^ Select validated si-RNA and Lipofectamine™ RNAiMAX at the concentrations selected from the optimization assay: 5 nM si-RNA (CAV1 silencing, Ambion, s2446), or 8 nM si-RNA (PAK1 silencing, Ambion, s10019) and 1 µl Lipofectamine/well. In parallel, a negative control si-RNA was used at the same concentration. Both si-RNA and Lipofectamine were diluted in Opti-MEM^®^ I Reduced Serum Medium, combined, mixed gently and incubated for 15 min at RT before being added drop-wise to the wells. All reagents were purchased from Thermo Fisher Scientific, Italy. After 24 h incubation the medium in the wells was exchanged with the standard cell culture medium containing 10% of serum and no antibiotics. 48 h after transfection the silencing efficiency was tested using TaqMan^®^ gene expression assay and the protein expression was evaluated by western blot method. The cell uptake of Rubipy-SiO_2_ NPs was quantified by flow cytometry after 3 and 6 h exposure in cells transfected with silencing-RNA in comparison to the cells transfected with negative control si-RNA.

### Real-time PCR amplification

Following transfection cells were washed with cold PBS and lysed in 350 μl RLT lysis buffer (Qiagen, Germantown, MD, USA). Samples were stored at −80 °C until RNA extraction was carried out. RNA was extracted from cells and purified using the RNeasy Plus kit (Qiagen, Germantown, MD, USA). The RNA quantification was done by a ND-1000 UV–Vis Spectrophotometer (NanoDrop Technologies), and the RNA integrity was assessed with the Agilent 2100 Bioanalyzer (Agilent), according to the manufacturer’s instructions. All RNA samples used in this study had a 260/280 ratio above 1.9 and a RNA Integrity Number (RIN) above 9.0.

1 µg of total RNA was reverse transcribed using the high-capacity cDNA Reverse Transcription Kit (Thermo Fisher Scientific, Italy), following the manufacturer's protocol. Real-Time PCR was performed with a total of 10 ng of cDNA for each reaction, using TaqMan^®^ gene expression assay for CAV1 (Hs00971716_m1, Applied Biosystems), for PAK1 (Hs00945621_m1, Applied Biosystems) for GAPDH as a negative control (Hs0275899_g1, Applied Biosystems) and TaqMan^®^ Universal PCR Master Mix (Applied Biosystems). The reaction was performed on Applied Biosystems 7900HT Fast Real-Time PCR System, with standard mode thermal cycling conditions, according to the manufacturer’s instructions. Analysis of real-time PCR data to quantify gene expression level was done by comparative Ct methods [14].

### Western blot

Following transfection (48 or 72 h) or exposure to Rubipy-SiO_2_ NPs (6 h), cells were rinsed twice with cold PBS and incubated with ice-cold lysis buffer (20 mM Tris–HCl, 100 mM NaCl and 1 mM EDTA, 0.5% Triton X-100) containing protease inhibitors cocktail (all from Sigma-Aldrich, Italy). After 10 min incubation on ice to ensure complete lysis, cell lysates were scrapped and centrifuged at 18,000×*g* for 15 min at 4 °C. The supernatant containing the cytoplasmatic protein fraction was transferred to a new tube. Protein concentration was measured by Bicinchoninic acid assay (BCA kit, Sigma-Aldrich, Italy). Equal amount of protein extracts (20 µg) were loaded onto a 10% SDS–polyacrylamide gel electrophoresis (SDS-PAGE) (Mini-PROTEAN^®^ BIORAD). Separated proteins were transferred to a methanol-activated Hybond-P membrane (Amersham Biosciences, USA) (Mini Trans-Blot BIORAD^®^). The PVDF membrane was probed with a primary rabbit polyclonal antibody against clathrin heavy chain (Abcam, 1:1000), anti-caveolin-1 (Abcam, 1:800), anti-PAK1 (Prestige Antibodies, Sigma-Aldrich, 1:250), anti-SNX5 (Abcam, 1:1000) or anti-GAPDH (Millipore Cat MAB374, Italy, 1:7500) as loading control. The membrane was then incubated with the secondary anti-rabbit (Sanzta-Cruz, 1:5000) or anti-mouse (Zymax antibodies, 1:3000) antibodies IgG-horseradish peroxidase-conjugated and detected by enhanced chemiluminescence (ECL, Amersham Biosciences, USA).

### Fluorescence microscopy

CaCo-2 cells were seeded at a density of 10^5^ cells/well in 4-chamber slides (Falcon), grown for 24 h and left untreated or incubated with chlorpromazine 50 µM, dynasore 80 µM, methyl-beta-cyclodextrin 5 mM, nystatin 40 µg/ml, genistein 200 µM, or EIPA 75 µM for 1 h at 37 °C. To investigate the energy dependence of NP uptake, CaCo-2 cells were exposed to 200 μg/ml of 30 and 80 nm-sized fluorescent Rubipy-SiO_2_ NPs for 3 h at 37 or 4 °C in complete cell culture medium.

Following exposure, cells were washed 3 times in PBS, fixed with 4% (v/v) paraformaldehyde in PBS and permeabilised with 0.1% (v/v) Triton X-100 in PBS (Sigma-Aldrich, Italy) before staining with AlexaFluor 488-conjugated Phalloidin (Thermo Fisher Scientific, Italy), diluted 1:100 for 40 min at RT. The nuclei were counterstained with the Hoechst 33342 dye (Dako, Italy). After staining, the cells were washed in PBS and mounted for microscopy. Images were acquired with an Axiovert 200 M inverted microscope equipped with a ApoTome slide module and Axiovision 4.8 software (Carl Zeiss; Jena, Germany), using a 40×/1.0 objective lens.

### Evaluation of cell metabolic activity (MTT assay)

Cells were grown in 96-well cell culture plates (Costar) until 75% confluent, exposed to Rubipy-SiO_2_ NPs for 48 h or to chemical inhibitors for 3.5 h and then washed in PBS. Cell viability was evaluated using MTT [3-(4,5-dimethylthiazol-2-yl)-2,5-diphenyl-2H tetrazolium bromide] (Sigma-Aldrich, Italy) added to the cells in fresh complete culture medium at a 250 μg/ml final concentration. After 2 h of incubation at 37 °C the supernatant was removed, the precipitated formazan crystals were dissolved in 0.1 M HCl in propan-2-ol and the absorbance was quantified at 540 nm in a multiwell plate reader (FluoStar, Omega, BMG Labtech, Offenburg, Germany). In parallel, to evaluate the possibility of interference of NPs with the assay, the PBS washing containing the silica NPs residues from each well was transferred to empty wells, incubated with MTT reagent in the conditions of the experiment and after 2 h the absorbance at 540 nm was read in a multiwell plate reader.

## Results

### Characterization of the size distribution and agglomeration state of Rubipy-SiO_2_ NPs

**Fig. 1:**
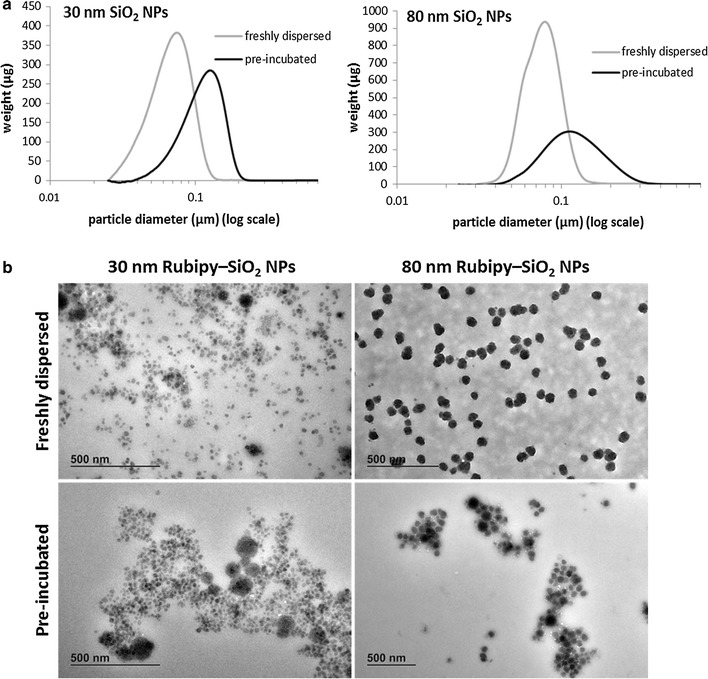
Size distribution of Rubipy-SiO_2_ NPs in complete cell culture medium. Rubipy-SiO_2_ NPs were suspended in CaCo-2 complete cell culture medium (10% of serum) at concentration of 1 mg/ml and the size distribution was evaluated either immediately or after 24 h pre-incubation at 37 °C by CLS (**a**) and by TEM (**b**)

Amorphous, negatively charged fluorescent Rubipy-SiO_2_ NPs of 30 and 80 nm were synthetized and characterized in water, PBS and cell culture medium as described previously [7]. The size distribution of Rubipy-SiO_2_ NPs in the complete CaCo-2 medium was measured by CLS immediately after preparing the NP suspension and after 24 h incubation at 37 °C (Fig. 1a; Table 1). In case of freshly prepared NP suspensions we observed a narrow size distribution of 80 nm NPs and a slightly larger peak of 30 nm NPs, indicating the initiation of the agglomeration already at this point. After 24 h incubation in the complete medium the size distribution has become much larger, and the average size of the particles similar for both types of Rubipy-SiO_2_ NPs. Moreover, visual inspection of both suspensions indicated agglomeration, and precipitation was visible to the naked eye.

**Table 1:** Average size and polydispersity of Rubipy-SiO_2_ NPs in water and complete cell culture medium measured by CLS (by weight), reported in nm

	H_2_O	Complete CaCo-2 medium (immediately)	Complete CaCo-2 medium (24 h incubation)
30 nm Rubipy-SiO_2_ NPs	26.1 ± 11	84.5 ± 64	129 ± 92
80 nm Rubipy-SiO_2_ NPs	68.8 ± 22	75 ± 49	116 ± 114

The CLS results were confirmed by TEM images (Fig. 1b) showing in fresh suspensions well dispersed 80 nm NPs and the 30 nm NPs initiating to agglomerate, and in suspensions incubated for 24 h the agglomerates of both sizes of Rubipy-SiO_2_ NPs together with the clumps of the proteins.

Therefore, taking into account the difference in agglomeration state and in order to facilitate the comprehension of the manuscript we will refer to the NPs incubated for 24 h in the complete medium as “agglomerated”, and to the NPs freshly suspended in the complete medium as “non-agglomerated”, even if NP agglomeration had already started in freshly prepared suspensions, particularly in those of 30 nm.

Fluorescence characteristics of Rubipy-SiO_2_ NPs were described in our previous study [7]. To ensure that the agglomeration process is not interfering with fluorescence measurement we carried out a scan of the fluorescence emission of both types of Rubipy-SiO_2_ NPs freshly dispersed in the complete Caco-2 medium and after 24 h incubation at 37 °C. No major changes in the fluorescence signal were noted with the increase of agglomeration state (Additional file 1: Figure S1), however the background fluorescence of the cellular medium was interfering slightly with the measurements. Since the intensity of fluorescence emission of 80 nm NPs was higher than the one of 30 nm NPs at the same mass concentration, we performed a calibration curve of the fluorescence intensity versus the mass concentration for each size of Rubipy-SiO_2_ NPs (Additional file 1: Figure S2) and we calculated the reference values that enabled the comparison of the cellular uptake of both sizes of Rubipy-SiO_2_ NPs (Additional file 1: Table S1).

### Cell uptake of agglomerated and non-agglomerated of Rubipy-SiO_2_ NPs

**Fig. 2:**
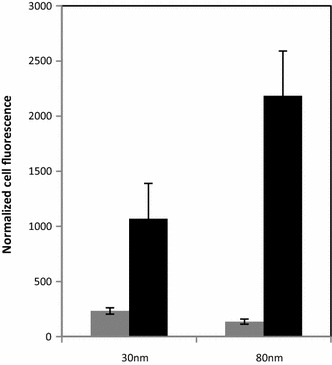
Effect of agglomeration state of Rubipy-SiO_2_ NPs on the level of cell uptake. The cellular uptake of Rubipy-SiO_2_ NPs was quantified by flow cytometry after 3 h exposure of CaCo-2 cells to 200 µg/ml of 30 and 80 nm Rubipy-SiO_2_ NPs either added to complete medium immediately before exposure (*grey bars*) or pre-incubated in complete medium for 24 h at 37 °C (*black bars*)

Rubipy-SiO_2_ NPs did not induce any toxicity to CaCo-2 cells for up to 48 h, as assessed by MTT assay (Additional file 1: Figure S3). For the quantification of the cellular uptake the cells were exposed to Rubipy-SiO_2_ NPs pre-incubated in the complete medium for 24 h and, in parallel, to Rubipy-SiO_2_ NPs freshly added to the complete medium, at the same concentration (200 µg/ml) and for the same exposure time (3 h). Measurements of the cell-associated fluorescence by flow cytometry, normalized to the fluorescence of NPs per mass unit, indicated a much higher cell uptake of the agglomerated form, particularly of 80 nm Rubipy-SiO_2_ NPs, than of the non-agglomerated form of NPs (Fig. 2).

**Fig. 3:**
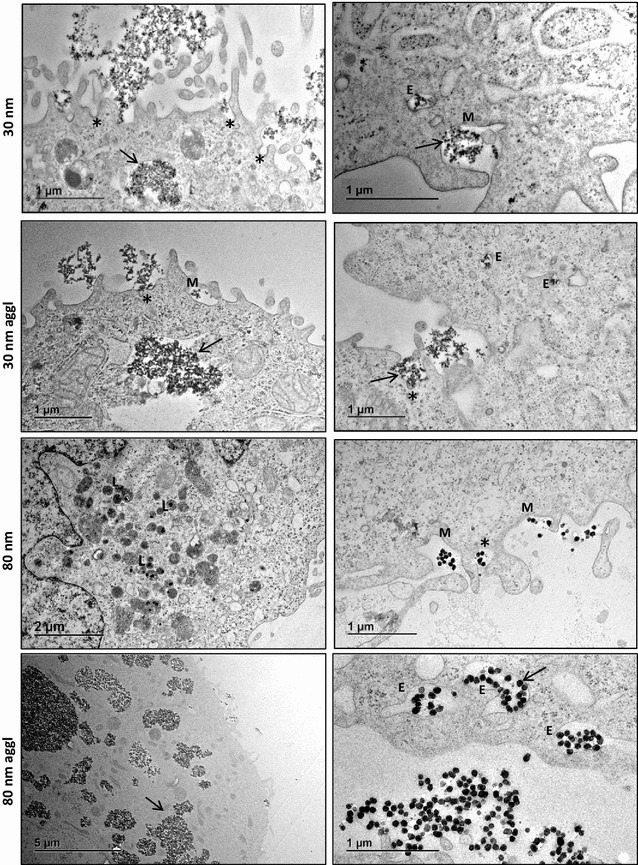
Cell uptake of Rubipy-SiO_2_ NPs observed by TEM. TEM images were obtained after 6 h exposure of CaCo-2 cells to 30 and 80 nm Rubipy-SiO_2_ NPs, either freshly added to cell medium or pre-incubated in the complete medium for 24 h (agglomerates); on the images we can observe clusters of NPs (*black arrows*), endosomes (*E*) and lysosomes (*L*) containing NPs, membrane ruffling typical for macropinocytosis (M), membrane invaginations (*Asterisk*). Note the flask-shaped endosomes on the *right-bottom image* (80 nm aggl)

TEM images were obtained after 3 and 6 h exposure to Rubipy-SiO_2_ NPs and showed an increased presence of NPs inside the cells after 6 h comparing to 3 h exposure (not shown). Both the agglomerated and non-agglomerated forms of 30 nm and the agglomerated 80 nm Rubipy-SiO_2_ NPs were observed as clusters, either interacting with the cellular membrane or in big endosomal vacuoles inside the cells (Fig. 3), whereas non-agglomerated 80 nm NPs were observed as small groups or single particles, thus showing a difference in the agglomeration state also at that level. The presence of many large clusters of 80 nm agglomerated Rubipy-SiO_2_ NPs inside the cells was particularly striking. Beside the large vacuoles, NPs were also observed in early and late endosomes (sometimes interestingly in flask-shaped endosomes) and lysosomes. In many areas and under all conditions it was possible to see characteristic ruffles of the plasma membrane involved in the macropinocytotic process (Fig. 3) or smaller invaginations of the membrane associated with clathrin- or caveolae-mediated endocytosis.

The evaluation of the kinetics of cellular uptake of Rubipy-SiO_2_ NPs was performed after 1, 3 and 5 h exposure at 37 and at 4 °C using flow cytometry (Additional file 1: Figure S4A). At 37 °C the interaction of the agglomerated 80 nm NPs with the cells was very intense up to 3 h and then reached a plateau, suggesting saturation, whereas the cell uptake of 30 nm Rubipy-SiO_2_ NPs was increasing proportionally to the time of exposure. Experiments carried out at 4 °C showed a strong inhibition of the cell uptake of Rubipy-SiO_2_ NPs indicating that the mechanism of the internalization was energy-dependent (Additional file 1: Figure S4 A, B). Still, a slight cell-associated fluorescence was detected in these conditions, though not increasing in time, suggesting that NPs were interacting with the cell membrane also at 4 °C.

### Effect of chemical inhibitors on cell uptake of Rubipy-SiO_2_ NPs

The use of chemical inhibition of the endocytic pathways, however widespread, is frequently criticised for lack of specificity, toxicity and multiple side effects [15, 16]. Consequently, a careful evaluation of the inhibitors’ effects on different endocytic pathways and for each tested cell line must be undertaken before actual experiments. Here, we used the classical markers of the endocytic pathways to assess the efficacy and the specificity of the employed inhibitors (Additional file 1: Figure S5): transferrin for clathrin-mediated endocytosis (CME), Bodipy-Lactosylceramide complexed to BSA (LaCer) for caveolae-mediated pathway and Dextran 10 kDa for macropinocytosis. We also evaluated the effect of the inhibitors on cellular morphology and on cell metabolism (Additional file 1: Figures S6, S7) to guarantee the absence of toxic effects on the cells in the range of the concentrations used in the study.

**Fig. 4:**
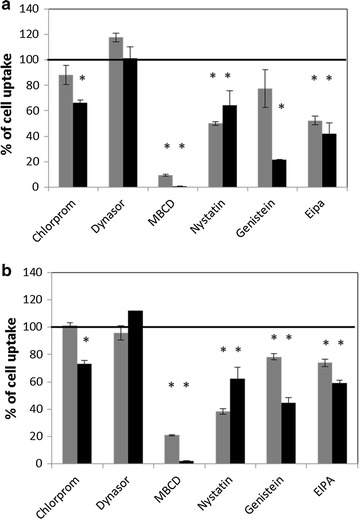
Inhibition of cell uptake of Rubipy-SiO_2_ NPs by endocytosis inhibitors. CaCo-2 cells were treated with the endocytosis inhibitors for 30 min, then exposed to 30 nm (**a**) and 80 nm (**b**) Rubipy-SiO_2_ NPs either freshly added to cell medium (*grey bars*) or pre-incubated in the complete medium for 24 h (*black bars*). The cellular uptake of NPs was quantified after 3 h exposure by flow cytometry and calculated as a percentage of the cell uptake in absence of the inhibitors, evaluated in parallel. Statistical significance was assessed by a Student’s *t* test. *p < 0.05, treated cells compared to untreated cells

Fluorescence of the cells exposed to Rubipy-SiO_2_ NPs after the treatment with the endocytosis inhibitors was compared to the fluorescence of the cells not pre-treated with the inhibitors. The inhibitors of CME did not decrease the uptake of Rubipy-SiO_2_ NPs, except a slight but significant effect of chlorpromazine on the uptake of agglomerated NPs (Fig. 4). This inhibitor was shown to act strongly and specifically on the CME (Additional file 1: Figure S5). Methyl-β-cyclodextrin (MβCD), which is inducing cholesterol depletion in the lipid rafts, blocked the cell uptake of Rubipy-SiO_2_ NPs almost completely and was the most potent among the tested inhibitors. However, it is not a specific inhibitor, acting on both caveolae-mediated endocytosis and macropinocytosis pathway (Additional file 1: Figure S5). Nystatin, a specific inhibitor of caveolae-mediated pathway was more efficient for non-agglomerated form of NPs (both 30 and 80 nm, ~40–50% remaining cell uptake) than for their agglomerated form (~60% remaining cell uptake). The opposite effect was observed after the treatment with genistein, which reduced the uptake of agglomerated 30 nm Rubipy-SiO_2_ NPs by almost 80%, while of non-agglomerated form of these NPs only by around 20%. However, in our experiments with endocytosis markers we observed the lack of specificity of this inhibitor, since it was acting on all three tested pathways (Additional file 1: Figure S5). Similarly, also ethyl-isopropyl amiloride (EIPA), frequently used to inhibit macropinocytosis, was shown to be non-specific. In our study, EIPA demonstrated a higher efficacy in the reduction of cell uptake of 30 nm NPs than 80 nm NPs, no matter what their agglomeration state was (Fig. 4).

### Colocalisation study and protein expression

**Fig. 5:**
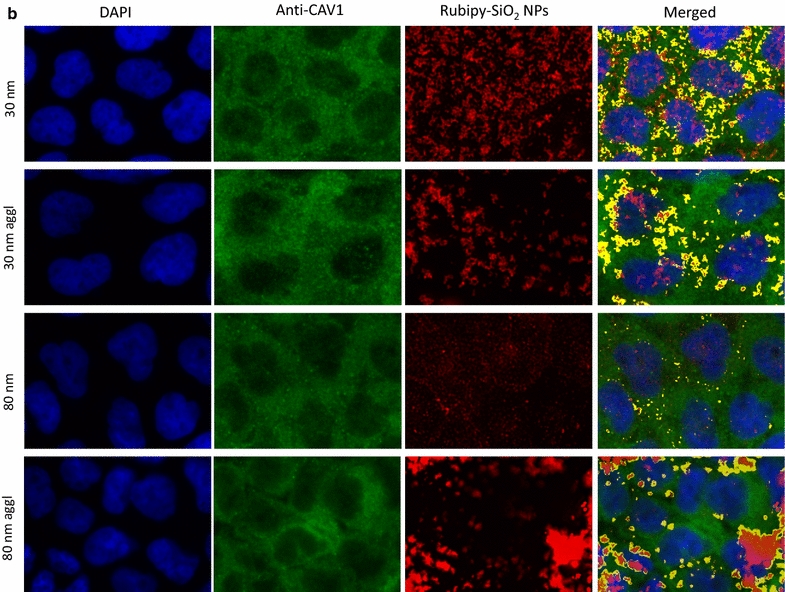
Colocalisation of Rubipy-SiO_2_ NPs with the CAV1 protein. After 3 h exposure to Rubipy-SiO_2_ NPs the cells were fixed and incubated with anti-CAV1. The images of different channels were merged and the colocalisation of signal from channel Cy3 (Rubipy-SiO_2_ NPs, shown in *red*) and of signal from channel Cy5 (secondary antibodies, shown in *green*) obtained by ImageJ is shown in *yellow*

**Fig. 6:**
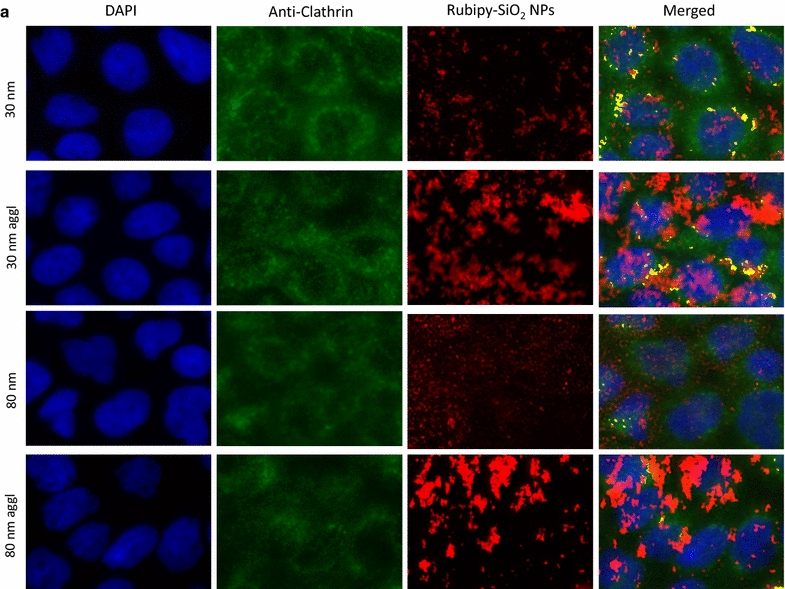
Colocalisation of Rubipy-SiO_2_ NPs with clathrin. After 3 h exposure to Rubipy-SiO_2_ NPs the cells were fixed and incubated with anti-clathrin. The images of different channels were merged and the colocalisation of signal from channel Cy3 (Rubipy-SiO_2_ NPs, shown in *red*) and of signal from channel Cy5 (secondary antibodies, shown in *green*) obtained by ImageJ is shown in *yellow*

**Fig. 7:**
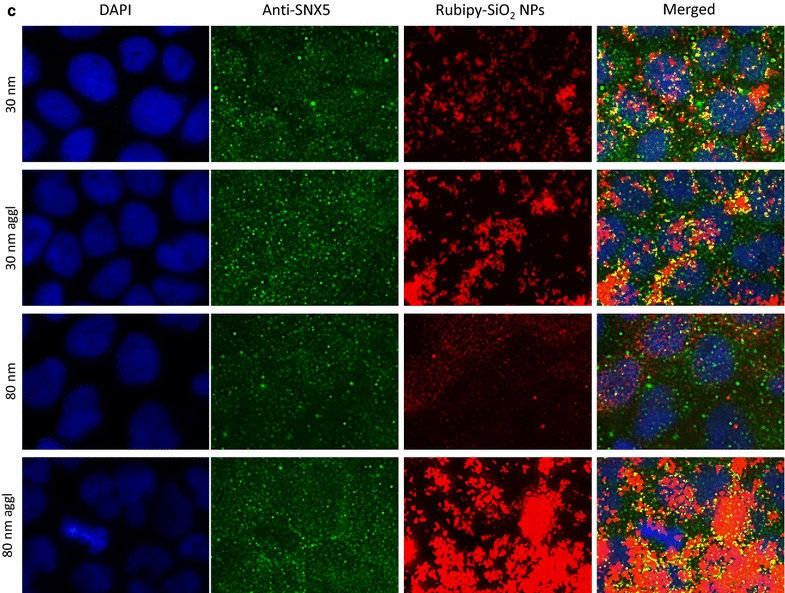
Colocalisation of Rubipy-SiO_2_ NPs with the SNX5 protein. After 3 h exposure to Rubipy-SiO_2_ NPs the cells were fixed and incubated with anti-SNX5 antibodies. The images of different channels were merged and the colocalisation of signal from channel Cy3 (Rubipy-SiO_2_ NPs, shown in *red*) and of signal from channel Cy5 (secondary antibodies, shown in *green*) obtained by ImageJ is shown in *yellow*

In the next step we investigated if Rubipy-SiO_2_ NPs internalized by the cells were colocalising with the proteins involved in different endocytic pathways: clathrin (for CME), caveolin-1 (CAV1, for caveolae-mediated endocytosis) and sorting nexin 5 (SNX5) involved in macropinocytosis. After 3 h exposure of Caco-2 cells to Rubipy-SiO_2_ NPs we observed that, independently of their size and agglomeration state, NPs colocalised to a great extent with CAV1 (Fig. 5), even if the presence of 80 nm non-agglomerated NPs was rather modest. We have seen also some colocalisation with clathrin, mainly for 30 nm NPs, but at much lower degree than with CAV1 (Fig. 6). All forms of Rubipy-SiO_2_ NPs except non-agglomerated 80 nm NPs colocalised well also with SNX5 confirming the implication of macropinocytosis in the internalization process of agglomerated NPs (Fig. 7). However, the expression of proteins in exposed cells was not in complete alignment with these results (Additional file 1: Figure S8), showing only very slight increase of CAV1 in cells exposed to 80 nm Rubipy-SiO_2_ NPs and of SNX5 in all treated cells. The expression of clathrin-HC was slightly decreased after the exposure to 80 nm agglomerated NPs, whereas the expression of PAK1 was at the same level in all tested conditions.

### Gene silencing

The results of the studies using chemical inhibitors and of the colocalisation assay suggested that mainly two pathways are implicated in the cellular uptake of Rubipy-SiO_2_ NPs: caveolae-mediated endocytosis and macropinocytosis. In order to focus on these pathways using a more specific method we employed silencing-RNA (si-RNA) targeting sequences of CAV1 and of p21-activated kinase (PAK1) implicated in macropinocytosis.

**Fig. 8:**
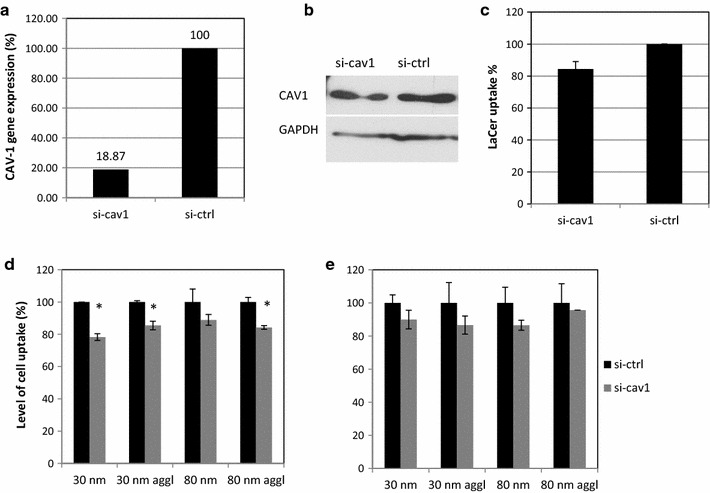
Inhibition of cell uptake of Rubipy-SiO_2_ NPs in CAV1-silenced cells. CaCo-2 cells were transfected with si-ctrl or si-cav1 and after 48 h the remaining RNA expression was evaluated with real-time PCR (**a**), protein expression was assessed with western blot (**b**), the cell uptake of LaCer was measured after 30 min incubation (**c**) and the cell uptake of Rubipy-SiO_2_ NPs was measured after 3 h (**d**) and 6 h (**e**) exposure by flow cytometry. Statistical significance was assessed by a Student’s *t* test; *p < 0.05, si-cav1 silenced cells compared to si-ctrl treated cells

The cells were first transfected with si-RNA targeting CAV1 (si-cav1) and with a si-RNA nonsense sequence used as a negative control (si-ctrl). 48 h after transfection RNA levels of the targeted gene were measured by the real-time PCR and the proteins expression was assessed by Western Blot. Despite a high rate of gene knock-down (>80%) (Fig. 8a) the depletion of CAV1 at the protein level was not very successful (Fig. 8b) since the protein was still present in the transfected cells, probably due to efficient recycling. Consequently caveolae-mediated internalization of LaCer was only slightly reduced compared to non-silenced cells (Fig. 8c). We evaluated the uptake of Rubipy-SiO_2_ NPs by the cells transfected with si-cav1 in comparison to the cells transfected with si-ctrl. However, we could observe only a slight reduction in the level of cell-associated Rubipy-SiO_2_ NPs after 3 h exposure and after 6 h exposure (Fig. 8d, e), suggesting that the partial depletion of CAV1 was not sufficient to effectively inhibit the endocytosis of NPs.

**Fig. 9:**
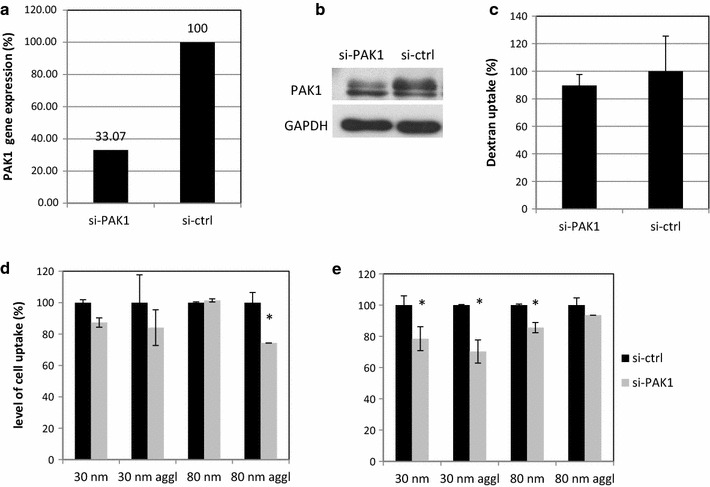
Effect of PAK1 depletion on cell uptake of Rubipy-SiO_2_ NPs. CaCo-2 cells were transfected with si-ctrl or si-PAK1 and after 48 h the remaining RNA expression was evaluated with real-time PCR (**a**), protein expression was assessed with western blot (**b**), the cell uptake of Dextran 10 kDA was measured after 30 min incubation (**c**) and the cell uptake of Rubipy-SiO_2_ NPs was measured after 3 h (**d**) and 6 h (**e**) exposure by flow cytometry. Statistical significance was assessed by a Student’s *t* test; *p < 0.05, si-PAK1 silenced cells compared to si-ctrl treated cells

The same procedure was used to evaluate the effect of PAK1 gene silencing. Here, 48 h after transfection the cells were demonstrating ~70% decrease in gene expression (Fig. 9a), and the depletion of PAK1 protein was not complete, but the upper band in the western blot was strongly reduced (Fig. 9b). Yet, this reduction was not sufficient to block the basal uptake of Dextran, which is usually used as a marker of the macropinocytosis (Fig. 9c). Indeed, depending on the cell line, incubation time and choice of analytical tool the internalisation of dextran was shown to be associated not only with macropinocytic process but also with CME, phagocytosis and other mechanisms [17, 18]. The uptake of the agglomerated 80 nm Rubipy-SiO_2_ NPs was significantly reduced in PAK1-silenced cells after 3 h exposure already, and after 6 h exposure the reduction of uptake was observed also for non-agglomerated 80 nm NPs and both forms of 30 nm NPs (Fig. 9d).

## Discussion

Silica NPs are finding multiple applications in nanomedicine and industry, as they are inexpensive, biocompatible, stable and easy to functionalize [19, 20]. In the area of drug delivery [21], but also food contact materials [22], the intestinal tract could be the first physiological barrier exposed to new nano-formulations. Even if perfectly stable in pristine conditions silica NPs can easily loose the colloidal stability in a new environment or in the presence of biomolecules. Here, we investigated how the agglomeration of silica NPs can impact on the interaction of NPs with the living cells. Our first observation was that the agglomeration of Rubipy-SiO_2_ NPs led to a major enhancement of their uptake by CaCo-2 cells, and that both agglomeration and cellular internalization were more pronounced for 80 nm than for 30 nm NPs. Similar findings, related to the increased cell uptake of agglomerated NPs, were already reported and explained by the modified kinetics of the deposition of the agglomerates on the cell layer compared to the monodispersed particles [23, 24].

However, in our study the agglomerates of silica NPs demonstrated not only modified kinetics but also a slightly modified mechanism of cellular uptake. Endocytosis is a complex process that can occur through several distinct but interdependent pathways, of which many are not well described yet [25, 26]. Here, we limited our research to the best studied pathways such as CME, caveolae-mediated endocytosis and macropinocytosis, but other clathrin- and caveolae-independent mechanisms could be involved as well in the internalisation process.

The clathrin-mediated pathway, the most extensively described pathway, is a receptor-mediated endocytosis. The involved proteins recruit cargo (approximately 100–200 nm) together with the receptor into developing clathrin-coated pits that are cut from the membrane invaginations via dynamin scission and form clathrin-coated vesicles. The individual vesicles can then fuse and form early endosomes, late endosomes and end up in lysosomes or can be recycled to the plasma membrane surface. CME is widely used for the specific uptake of certain substances required by the cell such as nutrients, antigens and growth factors including transferrin or LDL [27]. An upper size limit reported for particles entering via this pathway is 200 nm [8, 11]. Silica NPs of 200 nm and polymeric NPs of 100–200 nm were reported to internalize predominantly through CME [11, 28], and the positive charge on the surface of quantum dots [29], dendrimers [30] and polymer NPs [10, 31] was shown to increase the probability of internalization via CME rather than the use of other endocytic pathway. In our study, the inhibitors of CME were not very successful to inhibit the uptake of Rubipy-SiO_2_ NPs by CaCo-2 cells; however the effect of chlorpromazine and the colocalisation with clathrin suggests a minor role of this pathway in the internalization of 30 nm Rubipy-SiO_2_ NPs.

The caveolae-mediated pathway is characterized by the presence of small flask-shaped invaginations of the plasma membrane, responsible for uptake and transport of smaller (20–100 nm) molecules. Caveolae are lipid raft enriched and contain cholesterol-binding caveolins (mainly caveolin-1), essential for caveolae formation and function [26, 32, 33]. Once pinched off from the plasma membrane the caveolae vesicles transport and fuse with pH neutral caveosomes and are then transported to multivascular bodies, the Golgi apparatus or endoplasmic reticulum but not necessarily to acidic lysosomes [34]. Its potential to bypass the lysosomal degradation has been recently explored in nanomedicine as route for intracellular delivery of proteins and genes [35–37].

A negative surface charge of NPs has been found to trigger the cellular internalization predominantly via caveolae [10, 35, 38]. In agreement with these reports, also our study showed that the caveolae-mediated pathway was strongly implicated in the cellular internalization of the negatively-charged [7] Rubipy-SiO_2_ NPs. Colocalisation with CAV1 after 3 h exposure was obvious for all forms of NPs, and the CAV1 gene silencing, even if not very efficient at the protein level, induced a slight reduction of the cellular uptake of Rubipy-SiO_2_ NPs. Both immunofluorescence and gene silencing experiments indicated an abundant intracellular presence of CAV1 protein, which did not increase significantly upon exposure to Rubipy-SiO_2_ NPs. Interestingly, nystatin, which was a specific inhibitor of the caveolae-mediated pathway, decreased the uptake of non-agglomerated NPs more than agglomerated NPs, particularly those of 80 nm that according to characterization data displayed good monodispersity. MβCD was by far the most efficient to inhibit the uptake of all Rubipy-SiO_2_ NPs, however its mode of action based on the cholesterol depletion from the lipid rafts was not specific only to the caveolae-mediated pathway but also to other internalization mechanisms, mainly macropinocytosis (Additional file 1: Figure S5), a non-selective, clathrin and caveolae-independent endocytic mechanism.

Macropinocytosis is characterized by changes in the actin cytoskeleton and the membrane ruffles forming invaginations that enclose a region of extracellular fluid with suspended molecules [39, 40]. The internalized vesicles or macropinosomes have a size up to 5 µm, they undergo acidification and fuse with lysosomes. The non-specific inhibitor of micropinocytosis, EIPA, and other non-specific inhibitors in our study: MβCD and genistein, were more potent to block the internalization of agglomerated form than non-agglomerated form of NPs, suggesting the involvement of more than one pathway in the cell uptake of agglomerated NPs. The combination of endocytic pathways including the macropinocytosis was also suggested by TEM images showing characteristic macropinocytic ruffles of the plasma membrane accompanying the entrance of NPs but also small invaginations typical for the clathrin- or caveolae-mediated endocytosis. Study of the colocalisation of Rubipy-SiO_2_ NPs with SNX5 protein, involved in the formation of macropinosomes [39, 41], confirmed the involvement of macropinocytosis in the internalization of all forms of Rubipy-SiO_2_ NPs except 80 nm non-agglomerated NPs. Finally, we performed silencing of the PAK1 gene, an actin regulator shown to be required for both stimulated and basal fluid phase uptake [42]. After 3 h exposure to Rubipy-SiO_2_ NPs we observed a reduction in uptake of 80 nm agglomerated NPs by PAK1-silenced cells, and after 6 h the reduction of internalization of both forms of 30 nm of Rubipy-SiO_2_ NPs and of 80 nm non-agglomerated Rubipy-SiO_2_ NPs. This result was in agreement with the colocalisation study. As the process of internalization of agglomerated 80 nm Rubipy-SiO_2_ NPs was much faster in the control cells than in silenced cells and reached saturation after 3 h exposure (Additional file 1: Figure S4A), no difference in uptake between the silenced and control cells was visible after 6 h exposure. This fact points out the importance of selecting appropriate time-points in the study of the endocytosis process.

Macropinocytosis contributes to the internalization of bacteria, viruses, necrotic cells and larger particles, often in conjunction with other entry mechanisms [13, 43, 44]. However, smaller silica NPs of 20–100 nm were also shown to be internalized mainly by the macropinocytosis [45–47]. However, their size and state of agglomeration in complete medium was unfortunately not studied and it cannot be excluded that, similarly to Rubipy-SiO_2_ NPs, the initial small monodispersed NPs became large agglomerates in the presence of serum proteins.

## Conclusions

Taking into account the results of physicochemical characterisation of NP dispersion in the cell culture medium we demonstrated that the level of cell uptake and the mechanism of endocytosis of silica NPs were strongly dependent on their agglomeration state. Both caveolae-mediated endocytosis and macropinocytosis were implicated in the process of NP internalisation, whereas the clathrin-mediated pathway was involved to a minor extent. Well dispersed 80 nm Rubipy-SiO_2_ NPs were internalized mainly by the caveolae-mediated endocytosis, whereas 30 nm Rubipy-SiO_2_ NPs entered the cells via a combination of different endocytic pathways. Interestingly, with the increase of NP agglomeration we observed a highly enhanced cellular uptake and slightly modified mechanism of endocytosis with a predominant role of macropinocytosis. This finding highlights the importance of a careful evaluation of NP dispersion in relevant experimental conditions since a modified environment can easily induce NP agglomeration and consequently influence a biological response.

## Additional file

**Additional file 1.** Additional table and figures.

## Abbreviations

NPs: nanoparticles
DLS: dynamic light scattering
CLS: centrifugal liquid sedimentation
CCM: cell culture medium
TEM: transmission electron microscopy
CME: clathrin-mediated endocytosis
CAV1: caveolin-1
SNX5: sorting nexin 5
PAK1: p21-activated kinase
BSA: Bovine serum albumin
