# Supplementary material for: The agglomeration state of nanoparticles can influence the mechanism of their cellular internalisation
Source: J Nanobiotechnology. 2017 Jun 26;15:48. doi: 10.1186/s12951-017-0281-6 (PMC5485545; doi:10.1186/s12951-017-0281-6)

**The agglomeration state of nanoparticles can influence the mechanism of their cellular internalisation**

Blanka Halamoda-Kenzaoui, Mara Ceridono, Patricia Urban, Alessia Bogni, Jessica Ponti, Sabrina Gioria, Agnieszka Kinsner-Ovaskainen*

*European Commission Joint Research Centre, Directorate for Health, Consumers and Reference Materials, Via E. Fermi 2749, 21027, Ispra (VA)*

**Supporting Information**

**Fig. S1: Fluorescence spectrum of 30 nm and 80 nm Rubipy-SiO_2_ NPs in complete CaCo-2 medium.** Rubipy-SiO_2_ NPs were suspended in the complete CaCo-2 medium without phenol red at 100 µg/ml and the fluorescence scan (excitation 460 nm) was carried out immediately and after 24 h incubation at 37°C.

**
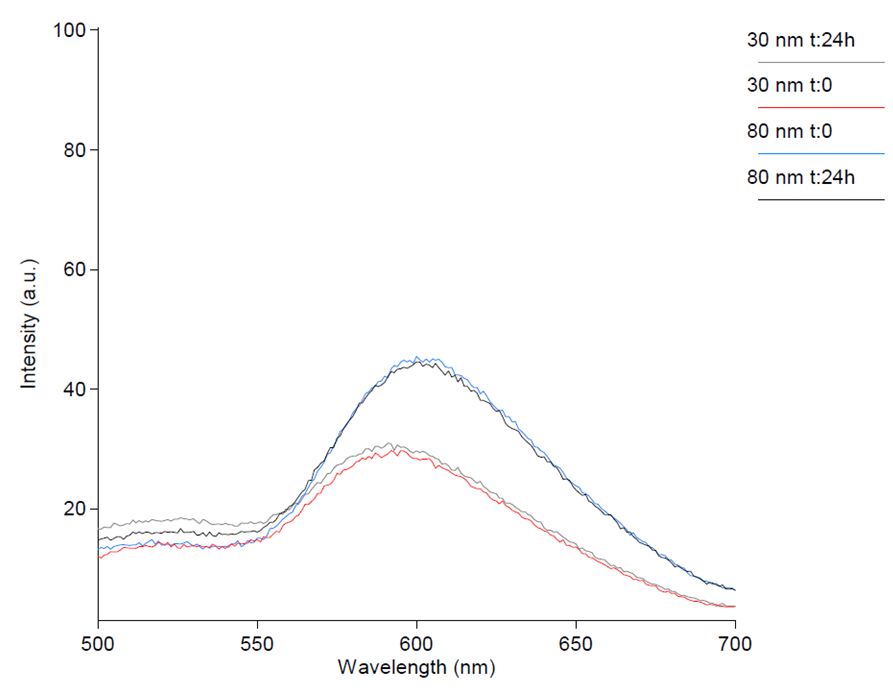
**

**Fig. S2**: **Calibration curve of the fluorescence intensity versus mass concentration for 30 nm and 80 nm Rubipy-SiO_2_ NPs measured by a fluorescence spectrophotometer*.***


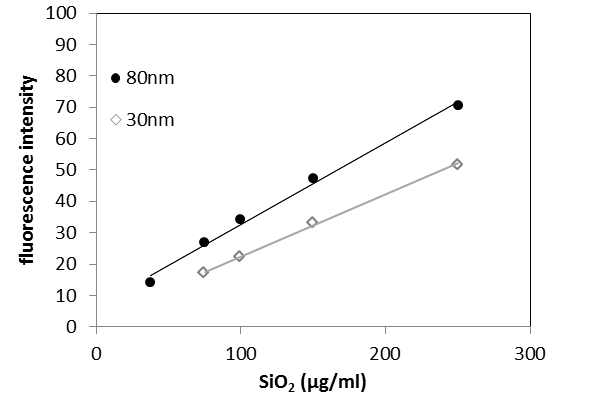


**Table S1: Calculation of relative fluorescence intensity per mass and per particle, based on the measurement of the fluorescence intensity of Rubipy-SiO_2_ NPs at 200 µg/ml in PBS by a spectrophotometer.**

|  | **Fluorescence intensity**  **at 200 µg/ml** | **Fluorescence intensity/µg** | **Number of NPs/ml** | **Fluorescence intensity/particle** |
| --- | --- | --- | --- | --- |
| 30 nm | 42.271 | 0.211 | 6.43E+12 | 6.57E-12 |
| 80 nm | 58.770 | 0.294 | 2.83E+11 | 2.08E-10 |

**Fig.S3: Effect of Rubipy-SiO_2_ NPs on the metabolic activity of CaCo-2 cells.**

Caco-2 cells were exposed to Rubipy-SiO_2_ NPs for 48 h and their metabolic activity was assessed by the MTT assay.

**
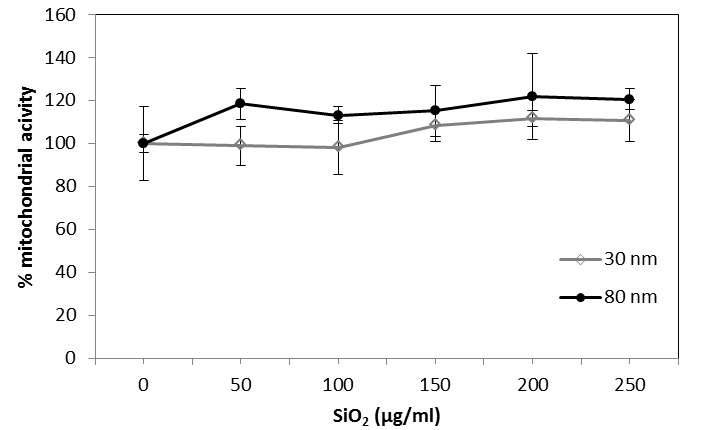
**

**Fig. S4: Effect of low temperature on the cellular uptake of Rubipy-SiO_2_ NPs.**

A: CaCo-2 cells were exposed to 200 µg/ml of 30 nm and 80 nm Rubipy-SiO_2_ NPs either freshly added (grey bars) or pre-incubated in complete medium for 24 h (black bars), and the cellular uptake of NPs was quantified by flow cytometry after 1 h, 3 h and 5 h exposure at 37°C or at 4°C.

B: CaCo-2 cells were exposed to 200 μg/ml of 30 nm and 80 nm Rubipy-SiO_2_ NPs in complete cell culture medium for 3 h either at 37°C or at 4°C, fixed and stained with AlexaFluor 488-conjugated phalloidin (actin filaments, green) and with Hoechst-33342 (nuclei; blue). Scale bars: 20 and 50 µm.


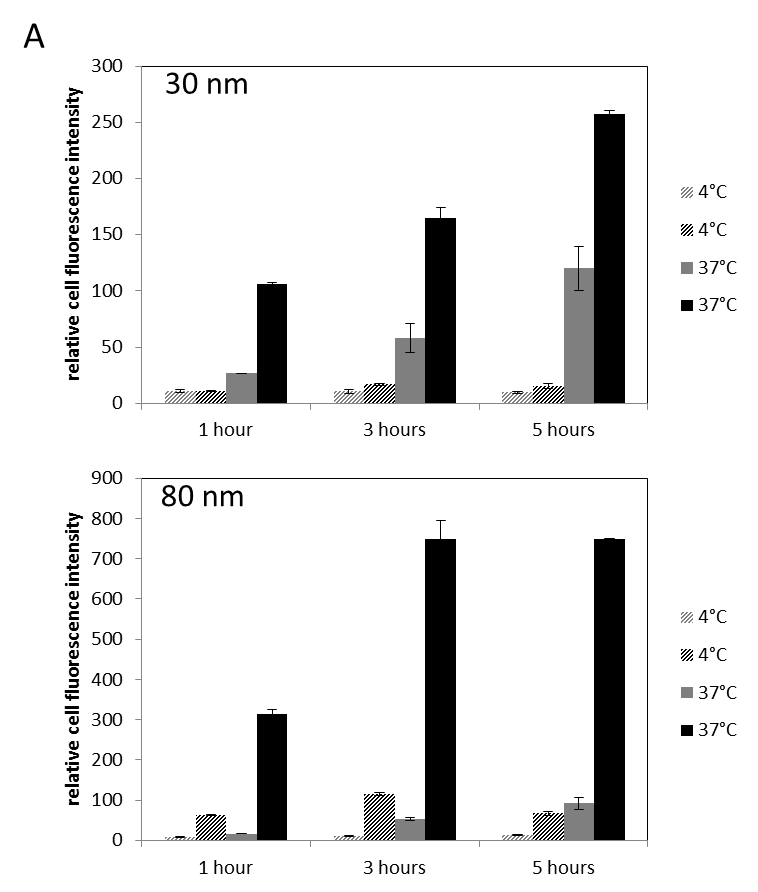


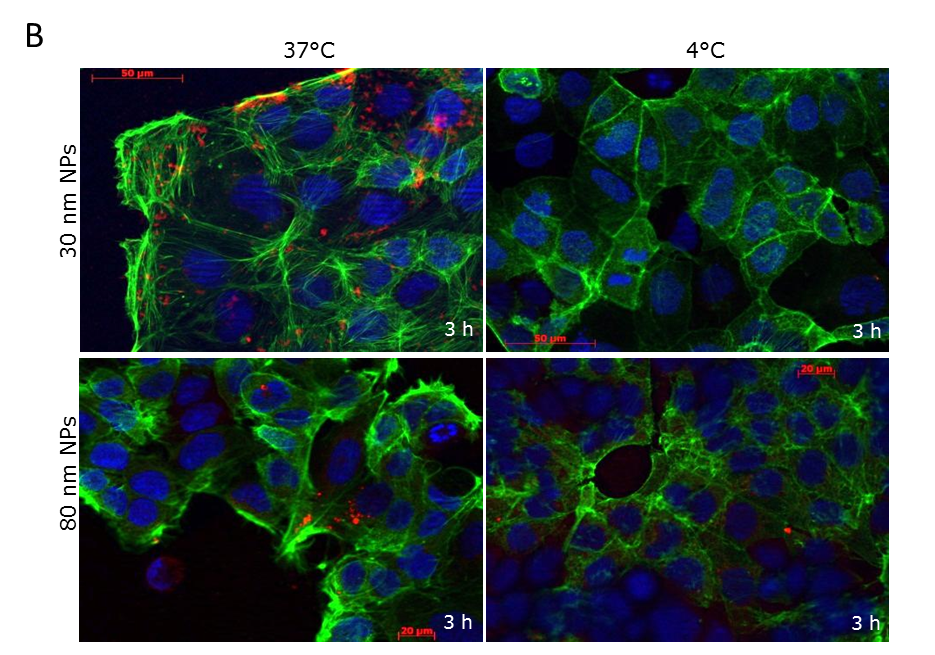


**Fig. S5: Evaluation of the efficacy and specificity of the chemical inhibitors.**

CaCo-2 cells were treated with the chemical inhibitors: chlorpromazine, MβCD, nystatin, EIPA and genistein for 30 min and then incubated with endocytosis markers: A: Transferrin (CME), B: LaCer (caveolae-mediated endocytosis), and C: Dextran 10 kDA (macropinocytosis) for another 30 min. The internalization of fluorescent endocytic markers was measured by flow cytometry and compared with their internalization without the pre-incubation with the inhibitors.


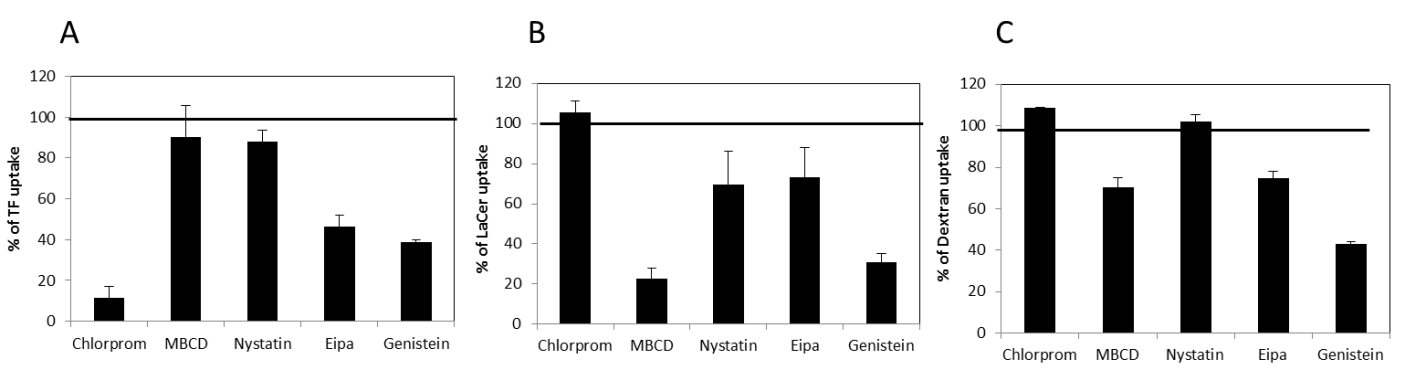


**Fig. S6:** **Actin morphology in inhibitor-treated CaCo-2 cells**

CaCo-2 cells were either untreated or treated for 1 h with different endocytic inhibitors, then fixed and stained with AlexaFluor 488-conjugated phalloidin (green). Nuclei were stained with Hoechst 33342 (blue). Scale bars: 20 and 50 µm.


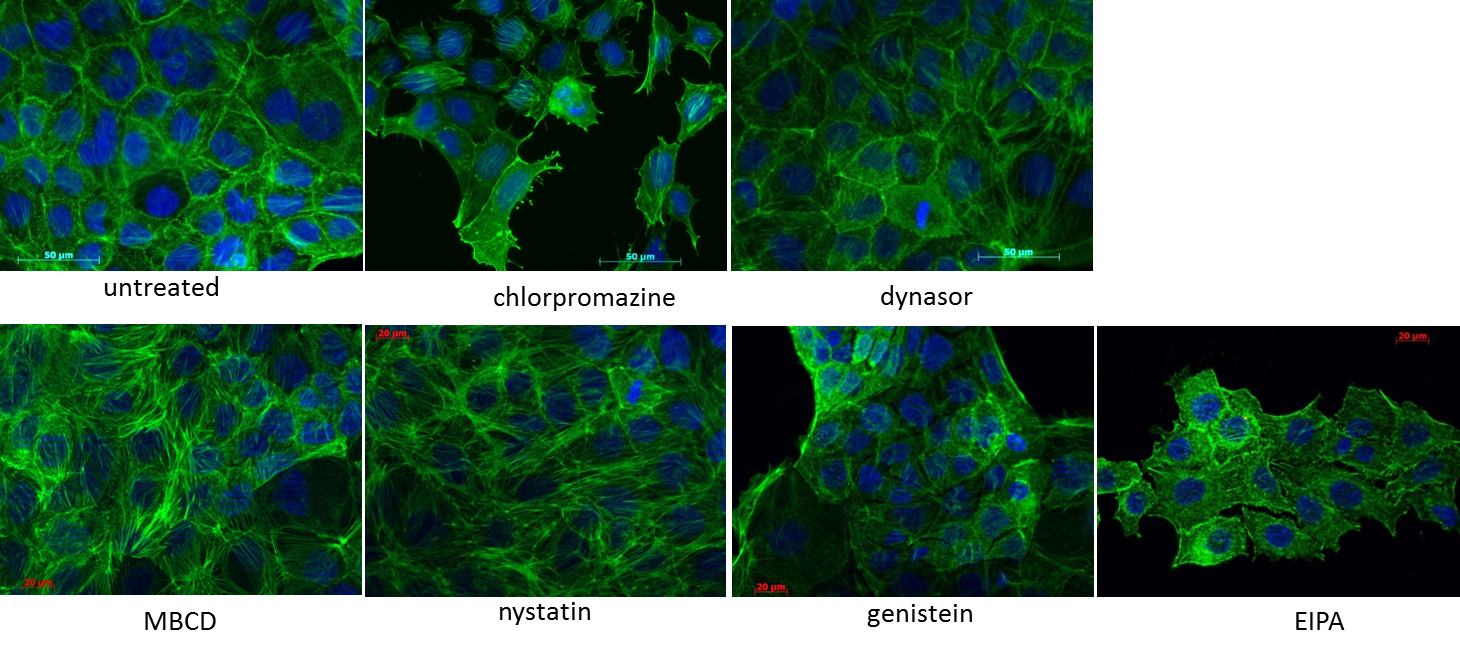


**Fig. S7: Effect of chemical inhibitors on the metabolic activity of CaCo-2 cells**

The MTT assay was performed after 3,5 h exposure of CaCo-2 cells to different concentrations of chemical inhibitors: chlorpromazin, MβCD, nystatin, genistein and EIPA.


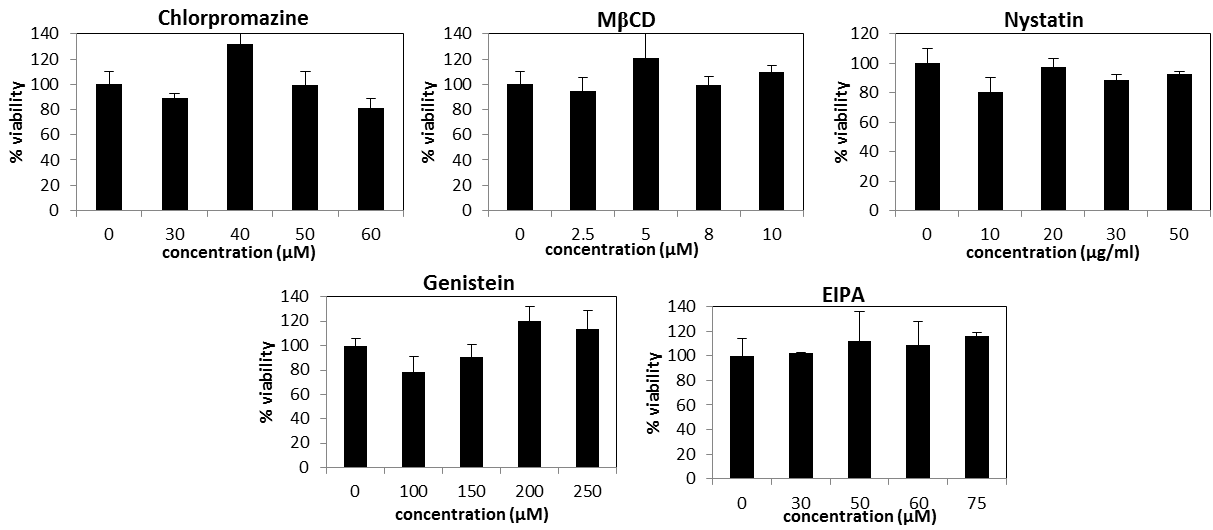


**Fig. S8: Expression of endocytic proteins by CaCo-2 cells exposed to Rubipy-SiO_2_ NPs.**

Following 6 h exposure to Rubipy-SiO_2_ NPs, CaCo-2 cells were lysed, and then the expression of either clathrin heavy chain, CAV1, SNX5 or PAK1 was determined by Western Blot in cell extracts. GAPDH expression was used as a loading control.


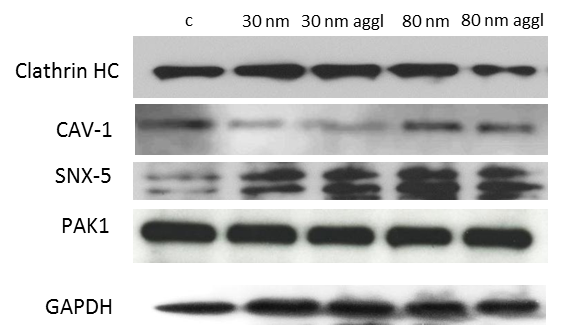

Supplement: Supplementary file 1 — Additional file 1. Additional table and figures. [file 12951_2017_281_MOESM1_ESM.docx]
